# Sex Differences in Athletic Performance Response to the Imagery and Mental Toughness of Elite Middle- and Long-Distance Runners

**DOI:** 10.3390/sports12060141

**Published:** 2024-05-23

**Authors:** Yunus Emre Yarayan, Serdar Solmaz, Mehdi Aslan, Alexios Batrakoulis, Sameer Badri Al-Mhanna, Kadir Keskin

**Affiliations:** 1 School of Physical Education and Sports, Siirt University, Siirt 56100, Turkey; yunus.emre.yarayan@gmail.com (Y.E.Y.); mhdiasln@hotmail.com (M.A.); 2 School of Physical Education and Sports, Batman University, Batman 72000, Turkey; serdarsolmaz11@gmail.com; 3 Department of Physical Education and Sport Science, University of Thessaly, 42100 Trikala, Greece; abatrakoulis@uth.gr; 4 Department of Physical Education and Sport Science, Democritus University of Thrace, 69100 Komotini, Greece; 5 School of Medical Sciences, Department of Physiology, Universiti Sains Malaysia, Kubang Kerian 16150, Kelantan, Malaysia; sameerbadri9@gmail.com; 6 School of Physical Education and Sports, Gazi University, Ankara 06560, Turkey

**Keywords:** athletic performance, imagery, mental toughness, athletics

## Abstract

This study aimed to determine whether there is a difference between the levels of imagery and mental toughness in the context of sports performance in male and female athletes. A total of 344 track and field athletes, 205 male (59.6%, 23.3 ± 4.0 years) and 139 female (40.4%, 22.9 ± 4.0 years), voluntarily participated in the study. Imagery Inventory and Mental Toughness Inventory in Sport were used as data collection tools in the study. In the evaluation of athletic performance, athletes were asked about their ranks in the years 2020, 2021, and 2022 and were categorized according to the scoring tables specified by the International Association of Athletics Federation. A MANOVA analysis was used to determine whether there was a difference between low (−2% to +5%), medium (+6 to +11%), and high (+12 to +17%) performers among male and female athletes, and a post hoc analysis was used to determine the source of the difference. According to the present findings, there was no significant difference between the imagery and mental toughness levels of athletes with high, medium, and low performance among male athletes. On the contrary, a significant difference was detected between the imagery and mental toughness levels of female athletes with medium and high performances, showing that athletes in the high-performance range had higher levels of imagery (Eta^2^ = 8) and mental toughness (Eta^2^ = 10) than athletes in the medium- and low-performance ranges. The findings of this study show that imagery and emotional intelligence are important factors for sports performance. In this context, coaches and sports psychologists can include these parameters in their training programs to achieve the optimal performance range.

## 1. Introduction

Since the beginning of human history, individuals, groups, and communities around the world have closely followed sporting events, have adopted athletes as exemplary personalities, and have seen these athletes as symbols representing themselves or their nations during the competitions they have watched [1]. In addition, transforming sport into a multi-billion dollar industry has increased the value placed on the achievements of countries, clubs, and athletes. Consequently, there is a growing need for a multidisciplinary approach to enhance athletes’ performance and success, pushing the boundaries of scientific knowledge in the process [2].

Athletic performance refers to the abilities, skills, physical and mental state, strategic and tactical approaches, technical competencies, and the capacity to achieve goals that an athlete or team exhibits in a particular sport [2,3]. Therefore, a multidisciplinary approach should be adopted to improve athletic performance [4,5].

Athletes are not only individuals with physiological and physical characteristics; they also have psychological, sociological, and cultural dimensions. These mental and emotional states play an important role [6]. Numerous researchers have highlighted the critical role of mental processes in shaping athletic performance and the essentiality of psychological prowess for attaining superior outcomes, underscoring the importance of developing mental and psychological processes such as motivation, concentration, imagery, and mental endurance [7,8,9,10,11,12,13,14]. This development is crucial for elevating sporting success from a certain level to higher echelons, linking the theoretical understanding of mental toughness and psychological strategies directly to practical applications in enhancing athletic performance [15,16]. Therefore, it is necessary to focus on developing mental and psychological abilities, as well as physical training [8,10,17,18,19,20].

These psychological factors affecting athletic performance are among the critical elements that determine the success of athletes [21]. These factors include athletes’ intrinsic motivation, self-esteem, stress coping skills, attention management, and mental toughness [22,23,24,25,26]. In the competitive environment of sports, mental toughness, the ability to cope with challenging conditions, can help athletes maintain their high-level performance and better cope with adverse situations [27]. Some researchers have defined mental toughness (MT) as a fixed trait that enables athletes to face and cope with all kinds of physical, mental, and emotional pressures, enabling them to reach an optimum level of performance. Effective management of these factors is essential for athletes to maximize their performance. Therefore, psychological skill development should be as important as physical training in training programs, and studies in this direction should be considered an important tool to ensure that athletes are psychologically ready at the highest level and to achieve peak performance [2,28,29,30].

In addition to mental toughness, imagery is one of the important techniques that aims to improve the psychological and mental performance and thus increase performance [31]. Imagery is often used in stress management and anxiety reduction techniques [32,33]. Imagery refers to a process in which people visualize their visual, intellectual, or conceptual experiences [34]. Individuals can think of different scenarios and mentally visualize these scenarios using their imagination. Imagery plays an important role as a mental preplanning process and creativity stimulus before acting [35]. In this context, imagery is a critical component of sports psychology and stands out as a strategy used by athletes to improve their performance. This method, also called mental training, allows athletes to use their brains to mentally visualize their future actions in advance. By doing so, athletes can motivate themselves more, improve their ability to cope with stress, and focus on their goals [33,36]. Studies in the literature have shown that researchers emphasize that imagery ability can support learning and performance, increase mental toughness by strengthening psychological characteristics, and is an important component of superior performance [9,31,35,37,38].

There are many differences between males and females in terms of physical and psychological factors. These differences are due to biological factors, hormonal differences, neurological structure, genetic differences, and differences in hormonal levels [39]. In addition, cultural and social norms, social expectations, gender roles, and social learning processes also differ [40]. In this respect, there are many differences between males and females, both physiologically and psychologically. Research on psychological and mental processes between the sexes also shows different results that make it difficult to draw a clear conclusion [41,42]. Biological differences are likely to affect coping with stress and emotional reactions through hormonal and biological structure [43], but there is no consensus on this issue. While experience and education levels may affect mental toughness, differences in stress coping strategies may be observed between different sexes [44]. Moreover, different motivations and goals according to sex may affect mental toughness levels [45]. When all these factors come together, it becomes difficult to draw a definitive conclusion about the differences in mental toughness between the sexes [46]. Further research and detailed investigations may provide a clearer understanding of this issue.

This study aimed to determine whether there is a difference between the levels of imagery and mental toughness in the context of sportive performance in male and female athletes to define and develop the concept of imagery and mental toughness, which is an important skill for success in sports. In this direction, it is thought that determining the differences related to the sportive performance parameter determined by concrete data specified by the International Association of Athletics Federations (IAAF) will contribute to the related literature. In line with this aim, the following hypotheses were formulated:

**H1:**  *There is a statistically significant difference in female and male athletes in terms of imagery level in the context of sportive performance.*

**H2:**  *There is a statistically significant difference in terms of mental endurance level in the context of sportive performance in female and male athletes.*

## 2. Materials and Methods

### 2.1. The Study Design

**Figure 1:**
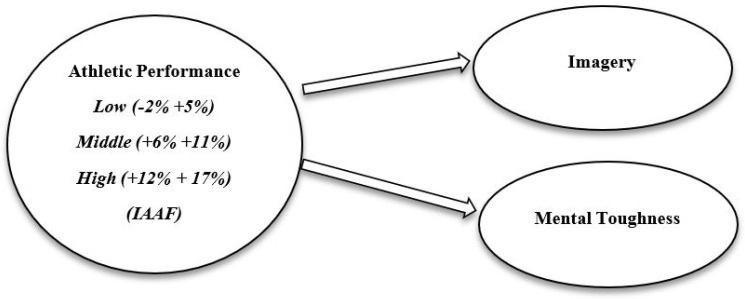
Study design model.

This research used a cross-sectional design [47,48]. Data were collected to determine the participants’ views on a topic or event or their characteristics, such as interest, skills, abilities, and attitudes [49,50]. The model is presented in Figure 1.

### 2.2. Participants

A total of 344 track and field athletes, 205 males (59.6%, 23.3 ± 4.0 years) and 139 females (40.4%, 22.9 ± 3.9), voluntarily participated in the study. All athletes were middle- and long-distance runners. The average sports experience of the participants was 9.8 ± 3.4 years for males and 9.8 ± 3.6 years for females.

### 2.3. Data Collection Process

First of all, to evaluate the ethical appropriateness of the research, the Siirt University Ethics Committee was involved, and ethics committee approval was obtained (21 February 2023: 523-4372). The data collection process continued for 4 weeks. During this process, 32 coaches were contacted, and positive feedback was received from 28 of them. In this context, information about the purpose of the study was provided by the responsible researcher on the appointment dates. The researchers first distributed a form created by themselves to the athletes. In these forms, the athletes were asked about their accomplishments in certain years, and the data were verified. In the next stage, imagery and mental endurance inventories were applied.

### 2.4. Data Collection Tools

#### 2.4.1. Personal Information Form

It consists of questions including demographic information about the sex, age, years of sport, and competition category experience of the athletes included in the study.

#### 2.4.2. Sports Performance Assessment

The scoring tables accepted by the IAAF were used to convert the average of the season best results of the athletes in 2020, 2021, and 2022 into a score and to determine their performance levels at the international level. The scoring tables were designed by Spiriev (2017) by statistically analyzing the competitive performances of track and field athletes worldwide [51]. The scores in the tables of different events cover equivalent performances. Therefore, the tables can be used to compare results obtained at different athletic events. Due to obvious biological differences, an exact comparison of the performances of men and women is not recommended. Therefore, the system includes separate scoring tables for men’s and women’s competitions, respectively. We collected performance times for three years and categorized the results based on the proportional changes in their performance levels. Specifically, we created categories to reflect whether the athletes’ performances had increased or decreased over the three-year period. The categories were defined as follows: low (−2% to +5%), medium (+6% to +11%), and high (+12% to +17%) performers. These category names were created to indicate the extent of the athletes’ performance changes over the three years. The scoring was based solely on the IAAF (International Association of Athletics Federations) scores [51].

To ensure accurate calculations, we used IAAF scores to evaluate the obtained performance results. This allowed us to compute the percentage changes in performance and place them into the appropriate category (low, medium, or high). Therefore, the categorization reflects the athletes’ percentage improvement or decline in performance over the three-year period, while the IAAF scores were used exclusively to score the achieved results.

#### 2.4.3. Mental Toughness Inventory in Sport

The “Sport Mental Toughness Questionnaire”, developed by Sheard, Golby, and Van Wersch (2009) to determine the level of mental toughness in a sport environment, consists of 14 items and 3 subdimensions [52]. The scale, which consists of three subdimensions (confidence, continuity, and control), as well as general mental toughness, has a 4-point Likert structure (1 = completely false; 4 = very true). The adaptation study of the Sport Mental Toughness Inventory (SZDE) into Turkish was conducted by Altıntaş and Bayar Koruç (2017). The results of the Cronbach’s alpha values determined for the subdimensions of the inventory are 0.84 for the confidence subdimension, 0.51 for the continuity subdimension, and 0.79 for the control subdimension. These results show that the scale is reliable [53]. In this study, the inventory was evaluated over the total score, and the internal consistency coefficient calculated for the inventory as a whole was found to be 0.91 for male participants and 0.82 for female participants.

#### 2.4.4. Imagery Inventory in Sports

The Imagery in Sport Inventory developed by Hall et al. (1998) to determine the level of imagery in a sport environment consists of 30 items and 5 subdimensions [32]. The adaptation study into Turkish was conducted by Kızıldağ and Tiryaki (2000). In the adaptation study, besides the general imagery level, the inventory consists of 21 items and 4 subdimensions (cognitive imagery, motivational specific imagery, motivational general—awareness, motivational general—skill). The inventory has a 7-point Likert scale (1 = strongly disagree; 7 = strongly agree) [54]. Within the scope of this study, the inventory was evaluated over the total score, and the internal consistency coefficient calculated for the overall inventory was found to be 0.93 for male participants and 0.94 for female participants.

### 2.5. Analysis and Interpretation of Data

The SPSS 22.0 (Statistical Package for Social Sciences, Chicago, IL, USA) computer program was used to analyze the data obtained from the data collection tools. In the data collected within the scope of the research, firstly, the control of the assumptions and the evaluation of the outlier analyses were made by considering the Mahalanobis distance. As a result of this process, it was determined that 7 people were considered outliers and 12 more people who answered the attention check questions in the inventories incorrectly. Based on the literature review, it has been stated that there are techniques such as assigning a value or excluding it from the analysis [55,56]. In this context, considering the number of the research group and the lack of answers to the inventory items, it was decided to exclude these data from those 19 people.

Accordingly, in the analyses performed on the data of the remaining 344 participants, the data were first checked with the Kolmogorov–Smirnov test, and it was determined that the data did not meet the normality assumptions. However, there are increasing opinions in the relevant literature that normality tests are not sufficient for Likert-type scales [57]. For this reason, the skewness kurtosis values that stand out for normality assumptions were examined, and it was determined that the distribution was normal [58]. In these tests, one-way MANOVA analysis was used to determine whether there is a difference between the imagery and mental toughness levels of male and female athletes (−2% +5%) for medium (+6% +11%) and high (+12% + 17%) performers. As a result of this analysis, the Bonferroni test, one of the post hoc tests, was used to determine the source of the difference. Within the scope of the research, the assumptions of the MANOVA analysis were checked. To conduct the MANOVA analysis, the variance and covariance matrices must be homogeneous. In this way, the homogeneity of the variance and covariance matrices was examined with the Levene F test and Box’s M test. Tabachnick and Fidell (2007) stated that Wilks’ Lambda λ value should be taken into account when the assumptions are met as a result of these analyses, and Pillai’s Trace value should be taken into account when the assumptions are not met [57].

## 3. Results

When the results of the MANOVA analysis according to the sportive performance variable in Table 1 were analyzed, it was determined that there was no significant difference in the integrated effect (Pillai’s Trace = 0.032; F = 1.621, *p* = 0.007). However, when the results of the total score of the inventories were examined, it was determined that there was no statistically significant difference in the variables of imagery (F = 2.225, *p* > 0.05) and mental toughness (F = 0.994, *p* > 0.05).

When the results of the MANOVA analysis according to the sportive performance variable in Table 2 were analyzed, it was determined that there was a significant difference in the integrated effect (Pillai’s Trace = 0.032; F = 1.621, *p* < 0.05). When the Eta Squared results were analyzed, it was seen that approximately 8% of the variance was explained by the independent variable. It was seen that the Eta Squared value obtained in the integrated effect was close to the medium level. However, when the results related to the total score of the inventories were examined, it was found that there was a statistically significant difference in the variables of imagery (F = 5.753, *p* < 0.05) and mental toughness (F = 7.510, *p* < 0.05).

When the results of the post hoc Bonferroni analysis to determine from which dependent variable the difference originated, it was seen that the imagery and mental toughness levels of the athletes with high-performance ranges were higher than the athletes with medium- and low-performance ranges. It was found that the variance explained for the imagery levels was 8% and the variance explained for mental toughness was 10%. It was seen that the related results were generally close to the average level.

## 4. Discussion

According to the results of our study, while there was no significant relationship between visualization and mental toughness skills and performance in male athletes, it was found that these skills were positively related to high performance among female athletes. This suggests that other factors may play a role in influencing performance in men, whereas women may benefit more from psychological preparation strategies. The finding suggests that sport psychology and performance enhancement strategies should be designed with gender differences in mind, suggesting that coaches and counselors should consider individual and gender-specific needs.

Day by day, studies investigating the relationship between sport performance and psychological skills are increasing in the sport psychology literature [31,59,60]. Considering that athletes have certain strategies for their performance, it is an undeniable fact that the right strategies are important factors that will make them successful [1]. Mental endurance and imagery are seen as one of the critical factors for success in sports performance [61,62].

For this purpose, by placing the findings of the study in a broader context, when various perspectives on the subject were examined, it was seen that there were both supportive and opposing views. Yadolahzadeh (2020), in a study conducted on female swimmers, found that imagery exercises improved swimming performance and stated that they can be used to improve swimming performance [63]. Similarly, an increase in sportive performance was observed in a group of taekwondo athletes who practiced imagery exercises [64]. In a study conducted by researchers on tennis players to evaluate the effect of this method, they suggested that imagery exercises should be included in training programs to increase motor performance, motivation, and sportive performance [65].

Most of the studies investigating the effect of imagery on sport performance have been conducted on athletes aged 18 years and older. However, Li-Wei, Qi-Wei, Orlick, and Zitzelsberger (1992), in their study on athletes between the ages of 7 and 10 playing table tennis, stated that imagery practices have an effect on improving shooting performance and thus sports performance in children, as well as in adults [66]. As a matter of fact, when we examine the relevant literature, it clearly shows the potential of imagery practices to increase sport performance [34,64,67,68,69,70,71,72,73,74]. In our results, it was observed that the imagery levels of athletes in the high-performance range were higher than those of athletes in the medium- and low-performance ranges and were in agreement with the literature. In conclusion, the evidence in the literature and our study results support the idea that imagery studies can have positive results in the field of sports and should be considered as a factor that increases sportive performance and should be taken into consideration when preparing training programs.

When we evaluate the opposing views, it is stated that the effect of imagery on sports performance may vary depending on athletes’ imagery skills, experience, and frequency of use [75]. Similarly, Cumming and Hall (2002) examined the effect of imagery training in athletes, and the results showed that the effect of imagery can vary depending on individual differences and how imagery skills are taught [76]. It has also been seen in studies in the literature that the effect of imagery on sport performance varies. These different results may be due to many factors, such as the characteristics of the sample groups used in the studies; methodological differences; or variables such as the disciplines, ages, gender, and experience levels of the athletes. In this context, we think that the link between imagery and sport performance varied according to sex in the results we obtained. Therefore, more comprehensive and detailed studies are needed to determine the specific effect of imagery on athletic performance.

Mental toughness, another psychological factor included in the scope of this research, stands out as a critical component for athletes to overcome difficulties, overcome failures, and perform at their best even under pressure. The impact of this capacity on athletes’ success is widely recognized in the sport psychology literature. However, the results of research on the extent and nature of this effect have not always been consistent. While some studies emphasize that mental toughness has a decisive role in sporting success, others state that this effect is valid under more specific conditions and circumstances [9]. In this section, when we evaluated the findings of the study in light of the existing literature, it was found that mental toughness contributes positively to performance and that cricketers develop this ability at the beginning of their careers in a study examining the mental toughness of English cricketers [77]. In a study on the mental toughness of the world’s top performing athletes, it was reported that this toughness is a critical element of high performance [9]. In the relationships between mental toughness, intelligence, and coping effectiveness, it has been found that athletes with high mental toughness use more effective coping strategies and therefore perform better [78]. Results from quantitative and qualitative studies on the importance of mental toughness suggest that it is a key component of high performance. Furthermore, the necessity of integrating methods that increase mental toughness into training has been emphasized [79]. In general, it has been reported that mental toughness is one of the most important psychological characteristics for achieving high performance [14,80,81,82,83,84,85]. As a result, the findings obtained within the scope of the research, together with the existing literature, support the idea that studies on mental toughness can positively affect sports performance and that this issue should be considered as an important factor in increasing sporting success. However, a review of the literature on mental toughness indicated that this topic is not fully elucidated and rather leads to conceptual confusion. According to the information available in the limited literature, there are no studies directly showing that mental toughness has no effect on sportive performance, but there are studies emphasizing that this relationship is complex [9]. In this context, 18 articles were examined in the study, in which the studies published on mental toughness and sportive performance between 2000 and 2020 were examined. The research results presented in 16 of the articles confirmed the positive correlation of mental toughness with sport outcomes and performance level. Only in equestrian and alpine skiing athletes was no such relationship found [81]. The relationship between mental toughness and sport performance is thought to vary due to factors such as differences between men and women, players in individual and team sports, the relationship between mental toughness and training experience, the ages of athletes, and the effectiveness of mental toughness programs.

## 5. Conclusions

This study revealed some differences between male and female athletes in terms of mental toughness and imagery abilities. Although it was determined that the mental toughness and imagery levels of individuals with high performance were higher in male athletes, this finding was not statistically significant. However, it was statistically proven that the mental toughness and imagery parameters evaluated in female athletes were at a better level in female athletes with high performance.

When these findings were evaluated, it may suggest that other factors affecting the performance of male athletes may be more dominant than mental toughness and imagery abilities. However, it can be stated that mental toughness and imagery levels play an important role in the performance of female athletes. Emotional, social, and cultural pressures on female athletes may have a more pronounced effect on the performance of mental toughness and imagery. This situation emphasizes the necessity for female athletes to use mental toughness and imagery effectively to improve their performance. In conclusion, this study shows that mental toughness and imagery abilities may have different effects on athletic performance depending on gender. This suggests that coaches and sports psychologists should adopt gender-specific approaches to optimize athletes’ performance. However, these results are limited to the present study and need to be confirmed by other large-scale studies.

## 6. Strengths and Limitations

In this study, the division of athletes into three different groups according to performance criteria contributes significantly to the clarity and comprehensibility of the study, thus allowing for more targeted observations rather than broad, generalized assessments. The research methodology, utilizing the International Association of Athletics Federations’ scoring system supported by robust empirical evidence, provides an objective and universally accepted approach to the assessment of athletic performance. This methodology is further refined through the use of gender-specific performance scoring tables that have been meticulously designed to neutralize the influence of biological differences between the sexes and thus ensure the accuracy of the results obtained.

However, the scope of the research, focusing predominantly on middle- and long-distance runners in track and field, places a potential limitation on the generalizability of the findings to athletes across a wider range of sports disciplines. This specialization may unintentionally narrow the applicability of the study’s results and thus raise questions about their relevance in other sports contexts. Furthermore, the study recognizes its limitations in accounting for the multifaceted nature of psychological variables such as imagery and mental toughness, which vary significantly between individuals. The exclusion of variables with the capacity to moderate external factors, such as training conditions, nutrition, and sleep quality, further highlights the limitations of the study’s comprehensive analysis and highlights areas for future research to explore in-depth our understanding of athletic performance in a wider range of contexts. We suggest future avenues of research involving other sports to discuss these limitations more thoroughly and to verify whether the observed trends are valid in different athletic disciplines.

## Figures and Tables

**Table 1:** MANOVA analysis results regarding the scores received from the Imagery and Mental Toughness Inventory of male athletes according to the sportive performance variables.

Inventories	Sportive Performance	N	$\bar{X}$	Sd	F	*p*	(η^2^)
Imagery Total	Low Performance (−2 +5)	55	71.10	15.86			
	Moderate Performance (+6 +11)	70	77.32	23.92	2.225	0.113	0.021
	High Performance (+12 +17)	80	77.72	17.20			
	Total	205	75.81	19.57			
Mental Toughness Total	Low Performance (−2 +5)	55	49.01	7.55			
	Moderate Performance (+6 +11)	70	49.10	9.25	0.994	0.372	0.010
	High Performance (+12 +17)	80	50.80	8.68			
	Total	205	49.74	8.60			

Box’s M *p* = 0.001; Pillai’s Trace = 0.032; *p* = 0.007; F = 1621; η^2^ = 0.016 ($\bar{X}$ = Mean, Sd = Standard Deviation).

**Table 2:** MANOVA analysis results regarding the scores received from the Imagery and Mental Toughness Inventory of female athletes according to the sportive performance variables.

Inventories	Sportive Performance	N	$\bar{X}$	Sd	F	*p*	(η^2^)
Imagery Total	Low Performance (−2 +5)	59	75.11	14.80			
	Moderate Performance (+6 +11)	46	80.19	13.64	5.753	0.004 *	0.078
	High Performance (+12 +17)	34	89.05	29.36			
	Total	139	80.20	19.73			
Mental Toughness Total	Low Performance (−2 +5)	59	46.74	8.36			
	Moderate Performance (+6 +11)	46	48.32	4.71	7.510	0.001 *	0.099
	High Performance (+12 +17)	34	51.97	3.00			
	Total	139	48.54	6.56			

Box’s M *p* < 0.001; Pillai’s Trace = 0.150; *p* < 0.001; F = 5510; η^2^ = 0.075 ($\bar{X}$ = Mean, Sd = Standard Deviation, * = Statistically Significant).

## Data Availability

The datasets used and/or analyzed during the current study are available from the corresponding author upon reasonable request.
